# Somatic, but not cognitive–affective, symptoms are associated with reduced heart rate variability in individuals with dysphoria

**DOI:** 10.3389/fpsyg.2015.00599

**Published:** 2015-05-07

**Authors:** Simone Messerotti Benvenuti, Giulia Buodo, Rocco Mennella, Daniela Palomba

**Affiliations:** ^1^Department of General Psychology, University of PadovaPadova, Italy; ^2^Center for Cognitive Neuroscience, University of PadovaPadova, Italy

**Keywords:** autonomic nervous system, cardiovascular risk, cognitive–affective symptoms, dysphoria, heart rate variability, somatic symptoms

## Abstract

**Background:** Somatic, but not cognitive–affective, symptoms of depression have been associated with reduced heart rate variability (HRV), and with poor prognosis in cardiovascular patients. However, factors concomitant with cardiovascular diseases may confound the relationship between somatic symptoms of depression and reduced HRV. Therefore, this study examined whether reduced HRV was differentially associated with cognitive–affective and somatic symptoms of depression in medically healthy individuals with and without dysphoria.

**Methods:** Self-reported cognitive–affective and somatic symptoms as measured with the Beck Depression Inventory-II questionnaire and time and frequency domain parameters of HRV were collected in 62 medically healthy individuals, of whom 25 with and 37 without dysphoria.

**Results:** Somatic, but not cognitive–affective, symptoms of depression were inversely associated with SD of NN intervals (β = -0.476, *p* < 0.05), number of interval differences of successive NN intervals greater than 50 ms (NN50; β = -0.498, *p* < 0.03), and HRV total power (β = -0.494, *p* < 0.04) in the group with dysphoria, after controlling for sex, anxiety, and lifestyle factors. Cognitive–affective and somatic symptoms were not related to any of the HRV parameters in the group without dysphoria (all *p*s > 0.24).

**Conclusion:** By showing that the relationship between somatic depressive symptoms and reduced HRV extends to medically healthy individuals with dysphoria, the present findings suggest that this association is independent of factors concomitant with cardiovascular diseases. The present study also suggests that individuals with somatic rather than cognitive–affective subsets of depressive symptoms may be at greater risk for developing cardiovascular diseases.

## Introduction

Depression is an important and independent risk factor for the onset of coronary artery disease (CAD) as well as for the adverse course and outcomes in patients with CAD (Wulsin and Singal, 2003; Barth et al., 2004) or following myocardial infarction (MI; Van Melle et al., 2004). Specifically, there is evidence that depression is associated with a 60% greater likelihood of having CAD (Wulsin and Singal, 2003). Patients with depression are also more likely to have a major cardiac event within a year of the diagnosis of CAD (Carney et al., 1988), and/or to die in the years following the diagnosis (Barefoot et al., 1996). Moreover, the presence of depression in post-MI patients is associated with a 2- to 2.5-fold increased risk of poor cardiovascular outcome (Van Melle et al., 2004). It has been also reported that depression is a risk factor for cardiac morbidity and/or mortality in patients who have undergone cardiac surgery (Lespérance et al., 2000; Blumenthal et al., 2003).

The autonomic nervous system (ANS) has been identified as a pivotal site of dysregulation, with reduced parasympathetic and/or increased sympathetic activity leading to arrhythmias and sudden cardiac death (Schwartz and Vanoli, 1981; Podrid et al., 1990; Musselman et al., 1998). Given that depression itself is associated with reduced parasympathetic and/or increased sympathetic activity (Kemp et al., 2010), the presence of depression is likely to potentiate the impaired parasympathetic control and increased sympathetic activity observed in patients with CAD. In turn, these depression-related ANS abnormalities may further predispose depressed patients with CAD to ventricular tachycardia, ventricular fibrillation, myocardial ischemia, and sudden cardiac death (Carney et al., 2002, 2005).

In line with these findings, reduced heart rate variability (HRV), which reflects excessive sympathetic modulation and/or inadequate cardiac vagal control, has been reported in psychiatric depressed individuals compared to healthy controls (Imaoka et al., 1985; Dallack and Roose, 1990; Rechlin et al., 1994). Importantly, there is even more evidence that depressed patients with CAD or after MI have reduced HRV compared to non-depressed individuals (Carney et al., 1995, 2001; for reviews, see Carney et al., 2002, 2005; Shaffer et al., 2014). It has been recently shown that an association between depression and reduced HRV extends also to patients who underwent cardiac surgery (Patron et al., 2012, 2014, 2015). Based on these findings, reduced HRV has been suggested as one possible pathophysiological marker of depression-related ANS dysregulation that has been implicated as a risk factor for cardiac morbidity and/or mortality in CAD, post-MI, or post-surgical patients (Carney et al., 2002, 2005; Patron et al., 2012, 2014, 2015).

It should be noted, however, that some studies failed to replicate the association between depression and reduced HRV in patients with CAD (Gehi et al., 2005) or post-MI (Martens et al., 2008). One possible explanation for these inconsistent findings may lie in the heterogeneity of depressive symptoms (Kemp et al., 2014), including cognitive–affective (e.g., negative self-image, guilt) and somatic (e.g., sleep problems, poor appetite, fatigue) symptoms. Indeed, a reanalysis of data from Gehi et al. (2005) showed that somatic, but not cognitive–affective, symptoms of depression were associated with reduced HRV in patients with stable CAD (de Jonge et al., 2007). Consistent with this finding, several lines of evidence have shown that cardiovascular prognosis is associated with somatic, but not cognitive–affective, depressive symptoms (e.g., Watkins et al., 2003; de Jonge et al., 2006; Linke et al., 2009; Roest et al., 2011). Specifically, Watkins et al. (2003) showed that medical comorbidity in patients with acute MI were more strongly associated with somatic than cognitive–affective depressive symptoms. Likewise, de Jonge et al. (2006) reported that somatic, but not cognitive–affective, symptoms of depression were related to poor cardiovascular prognosis in patients 2.5 years after MI, a finding that has been replicated by others (Smolderen et al., 2009; Martens et al., 2010; Roest et al., 2011). Notably, Linke et al. (2009) and Schiffer et al. (2009) have recently extended these findings to women with suspected myocardial ischemia and patients with chronic heart failure, respectively.

Despite the intriguing evidence suggesting that somatic, but not cognitive–affective, symptoms of depression may differentially impact cardiovascular risk by reducing HRV (de Jonge et al., 2007), research on depression and HRV has been typically conducted in cardiovascular patients (Kemp et al., 2010). Therefore, many factors concomitant with cardiovascular diseases (e.g., disease severity, the presence of comorbid medical conditions) may confound the observed association between somatic symptoms of depression and reduced HRV (Carney and Freedland, 2012). Another important condition that may impact the relationship between somatic symptoms and HRV in cardiovascular patients with depression is the presence of anxiety symptoms. Some studies have suggested that anxiety – a comorbid condition reported in more than 60% of patients with major depression disorder (MDD; Kessler et al., 2005) – rather than depression is associated with reduced HRV (Thayer and Lane, 2000; Cohen and Benjamin, 2006; Friedman, 2007; Rottenberg, 2007; Miu et al., 2009), and with the risk of cardiovascular diseases (Cohen and Benjamin, 2006; Vogelzangs et al., 2010). Consistent with these findings, Martens et al. (2008) have shown that anxiety, but not depression, was associated with reduced HRV. Surprisingly, despite this converging evidence, the majority of studies examining the relationship between depression and reduced HRV in cardiovascular patients did not control for the effects of anxiety (e.g., Carney et al., 1995, 2001; Stein et al., 2000; de Jonge et al., 2007).

In light of these considerations, it is crucial to examine whether HRV is differentially associated with subsets of depressive symptoms in medically healthy individuals, while controlling for anxiety symptoms. At present, only one study to our knowledge has examined whether symptom dimensions of depression were differentially related to HRV in medically healthy preadolescents (Bosch et al., 2009). Specifically, consistent with findings observed in patients with cardiovascular disease, Bosch et al. (2009) reported that somatic, but not cognitive–affective, depressive symptoms were inversely associated with HRV and baroreflex sensitivity. However, the results by Bosch et al. (2009) were limited to the preadolescent population, aged between 10 and 12 years. This limitation is even more important when one considers that HRV parameters reach their maximum at about 20 and 30 years of age (Korkushko et al., 1991), and differ between healthy individuals aged 10 years and those aged >16 years (Silvetti et al., 2001). In addition, Bosch et al. (2009) did not control for the potential effect of anxiety on the relationship between somatic depressive symptoms and reduced HRV.

Therefore, the aim of the present study was to examine whether somatic and cognitive–affective symptoms of depression would be differentially associated with time and frequency domain parameters of HRV in medically healthy individuals aged 18–30 years, while controlling for sex, anxiety, and lifestyle factors. Based on previous findings (de Jonge et al., 2007; Bosch et al., 2009), it was hypothesized that HRV parameters would be inversely related to somatic, but not cognitive–affective, symptoms of depression. Given that somatic, but not cognitive–affective, subsets of depressive symptoms are distributed taxonomically (Beach and Amir, 2003), it is possible that depression may influence HRV parameters only when somatic depressive symptoms exceed a certain threshold of severity. Therefore, it was also examined whether somatic or cognitive–affective symptoms of depression would be differentially correlated with HRV parameters in individuals with or without dysphoria. To this end, we divided the whole sample into two subgroups depending on the presence/absence of dysphoria. It was expected that somatic depressive symptoms would be inversely related to HRV parameters in individuals with, but not without, dysphoria.

## Materials and Methods

### Participants

After local Ethics Committee approval, 62 undergraduate students from the University of Padova were enrolled in the present study. Participants were mostly females (*n* = 54; 87%), with a mean (SD) age of 21.9 (2.0). None of the participants were taking antidepressants or medications known to affect ANS functioning or had a history of neurological or cardiovascular disease. The descriptive statistics are reported in **Table 1**.

**Table 1:** Characteristics of the participants enrolled in the study.

Variables	Participants (*n* = 62)
Female sex	54 (87)
Age (years)	21.9 (2.0)
Education (years)	15.8 (1.3)
Current smoker	24 (39)
Regular alcohol use	6 (10)
STAI-Y2	42.2 (12.5)
BDI-II	8.4 (7.1)
Cognitive–affective symptoms	4.4 (4.7)
Somatic symptoms	4.0 (3.1)
HR (bpm)	77.1 (11.8)
SDNN (ms)	54.2 (19.9)
rMSSD (ms)	42.5 (24.2)
NN50 count	74.7 (57.0)

*Data are *M* (SD) of continuous and *N* (%) of categorical variables. BDI-II, Beck Depression Inventory-II; HR, heart rate; bpm, beats per minute; SDNN, standard deviation of NN intervals; rMSSD, the square root of the mean of the sum of the squares of differences between adjacent NN intervals in ms; NN50, number of interval differences of successive NN intervals greater than 50 ms*.

### Assessment of Depressive and Anxiety Symptoms

Depressive symptoms were assessed using the Beck Depression Inventory-II (BDI-II; Beck et al., 1996; Ghisi et al., 2006). The BDI-II is a reliable and valid self-report questionnaire that evaluates the severity of symptoms of depression in the past 2 weeks. Answers are given on a four-point (0–3) Likert scale and scores range from 0 to 63, with higher scores indicating more severe depressive symptoms. Of note, there is large evidence that the BDI-II is a reliable and valid measure of the severity of depressive symptoms in community samples (Lasa et al., 2000) and college populations (Sprinkle et al., 2002). In the Italian version, a score of 12 has been identified as an optimal cut-off to discriminate individuals with and without problems of depression in the Italian population (Ghisi et al., 2006).

The structure of the BDI-II has been examined in several studies using factor analysis (Ward, 2006). A two-factor solution has been frequently reported in non-clinical (Whisman et al., 2000; Storch et al., 2004) and clinical samples (Beck et al., 1996; Steer et al., 1998, 1999). Specifically, the two-factor solution usually comprises a cognitive–affective factor and a somatic factor in non-clinical individuals (mostly students; Beck et al., 1996; Whisman et al., 2000; Storch et al., 2004; Ward, 2006) as well as in psychiatric patients (Beck et al., 1996; Steer et al., 1998, 1999). Separate scores for each factor were calculated for each participant.

The module A of the Structured Clinical Interview (SCID-I) for the DSM-IV Axis I (First et al., 1997; Italian version by Mazzi et al., 2000) was administered in order to confirm the presence of dysphoria (i.e., at least two current symptoms of depression; Judd et al., 1994; Maier et al., 1997) and to exclude individuals with major depression, dysthymia, or bipolar disorder.

Self-reported trait anxiety symptoms were assessed using the State–Trait Anxiety Inventory, form Y2 (STAI-Y2; Spielberger et al., 1996). The STAI form Y1 and Y2 is composed by two 20-item four-point Likert scales that measure state and trait anxiety, respectively. State anxiety represents the individual’s fluctuant and transitory anxiety, whereas trait anxiety indicates long-lasting and persistent anxiety. Accordingly, test-retest reliability within a 1-month interval is very high for STAI-Y2 (*r* = 0.82), which evaluates the stable trait of anxiety, whereas it is moderate for STAI Y1 (*r* = 0.49), which, in turn, evaluates current and time-dependent anxiety. Raw scores range between 20 and 80 for both scales, with higher scores reflecting higher levels of trait or state anxiety. Given that reduced HRV is more likely to be associated with trait rather than state anxiety, we included only trait anxiety in the statistical analyses.

### HRV Recordings

The electrocardiogram (ECG) was recorded using Ag/AgCl surface electrodes positioned on the participant’s chest in a modified lead II configuration. The ECG signal was amplified with a gain of 150, bandpass filtered (0.3–100 Hz, to avoid distortions of the T-wave signal), digitized at 500 Hz (16 bit A/D converter; resolution 0.559 μV/LSB), and stored on to a Pentium IV computer. Impedance was kept below 5 kΩ. The ECG signal was amplified and filtered by a SynAmps unit amplifier (Neuroscan, Inc., Compumedics, Ltd, El Paso, TX, USA). A digital trigger detecting R-waves was applied to the ECG signal to obtain RR intervals, corresponding to the inverse of heart rate (HR). All ECG data were visually inspected and artifacts were corrected with a piecewise cubic spline interpolation method that generates missing or corrupted values into RR intervals. Then, time domain parameters of HRV were calculated by Kubios HRV Analysis Software 2.0 (The Biomedical Signal Analysis Group, Department of Applied Physics, University of Kuopio, Finland). Time domain parameters, which are measures of the total variation of HR, were calculated as follows:

(1) Standard deviation of NN intervals (SDNN) expressed in ms, which reflects the cyclic components responsible for the variability of HR (Task Force of the European Society of Cardiology, and the North American Society for pacing, and electrophysiology, 1996);
(2) Root mean square of successive difference of NN intervals (rMSSD) expressed in ms, which reflects estimates of short-term variability of HR. rMSSD is highly sensitive to the fluctuation of high frequency (HF) of HRV and is an index of cardiac vagal control (Task Force of the European Society of Cardiology, and the North American Society for pacing, and electrophysiology, 1996);
(3) Number of interval differences of successive NN intervals greater than 50 ms in the entire recording (NN50), which reflects estimates of short-term variability of HR and is an index of cardiac vagal control (Task Force of the European Society of Cardiology, and the North American Society for pacing, and electrophysiology, 1996).

Fast Fourier spectral analysis was then applied on the NN series to compute frequency domain indexes, which, in turn, were calculated as follows:

(1) HRV total power in ms^2^, which reflects the variance of all NN intervals (Task Force of the European Society of Cardiology, and the North American Society for pacing, and electrophysiology, 1996);
(2) HF power (0.15–0.40 Hz) in ms^2^, which primarily reflects cardiac parasympathetic tone (Task Force of the European Society of Cardiology, and the North American Society for pacing, and electrophysiology, 1996).

All frequency domain indexes were logarithmically transformed to normalize their distribution.

### Procedure

Upon arrival at the laboratory, the participants read and signed an informed consent form and completed the BDI-II and STAI-Y2 questionnaires. In order to confirm the presence of dysphoria and to exclude the presence of major depression, dysthymia, or bipolar disorder, all the participants were administered the SCID-I for the DSM-IV by a trained psychologist. The participants completed an *ad hoc* interview that provided information on their age, health status, smoking habits, and regular alcohol use. Then, each participant was seated in a comfortable armchair in a sound-attenuated, dimly-lit room. After the electrodes were attached, the ECG was continuously recorded at rest over a 5-min period. In order to avoid movement artifacts, participants were instructed to stay still and not to talk during the ECG recordings.

### Statistical Analysis

According to the two-factor structure by Carvalho Bos et al. (2009), cognitive–affective symptoms were calculated by summing the scores of 12 BDI-II items for each individual: indecisiveness, worthlessness, loss of pleasure, punishment feelings, past failure, pessimism, self-criticalness, sadness, loss of interest, self-dislike, guilty feelings, and suicidal thoughts. Somatic symptoms were calculated by summing the scores of the remaining nine BDI-II items: agitation, changes in appetite, crying, tiredness or fatigue, loss of energy, irritability, changes in sleeping pattern, concentration difficulty, and loss of interest in sex. Internal consistency measured by Cronbach’s alpha was high for both the cognitive–affective factor (Cronbach’s alpha = 0.88) and the somatic factor (Cronbach’s alpha = 0.78).

As our first step, a linear regression model was used to predict HR, SDNN, rMSSD, NN50, HRV total power, and HF power from the overall depressive symptoms (i.e., the total BDI-II score) with the group as a whole. Then, linear regression models were used to examine whether cognitive–affective and/or somatic symptoms were associated with HR, SDNN, rMSSD, NN50, HRV total power, and HF power.

As our second step, the whole sample was divided in two subgroups depending on the presence of dysphoria. In line with recommendations for the use of the BDI-II in non-clinical samples, and for categorization of research participants (see Kendall et al., 1987; Haaga and Solomon, 1993; Tennen et al., 1995; Frewen and Dozois, 2005), individuals who scored equal to or greater than 12 on the BDI-II, had at least two current depressive symptoms, at least 2 weeks in duration, and did not meet the diagnostic criteria for major depression, dysthymia, or bipolar disorder were assigned to the group with dysphoria (*n* = 25). The participants who scored lower than 12 on the BDI-II and had no current symptoms of depression or previous diagnosis of major depression, dysthymia, or bipolar depression were assigned to the group without dysphoria (*n* = 37). Chi-square or Fisher’s exact test or analyses of variance (ANOVAs) were used to compare the groups in terms of sex, age, smoking habits, regular alcohol use, cognitive–affective, and somatic symptoms, and anxiety symptoms. Linear regression models were used to examine whether overall depressive symptoms were associated with HR, SDNN, rMSSD, NN50, HRV total power, and HF power separately in the group with vs. without dysphoria. Then, linear regression models were used to predict HR, SDNN, rMSSD, NN50, HRV total power, and HF power from cognitive-affective, and somatic symptoms in both groups separately. Sex, anxiety symptoms, smoking habits, and regular alcohol use were included as covariates in each regression model. Age was not included as a covariate in the regression models due to the small age range of participants (SDs ≤ 2.0). In addition, all the participants were between 19 and 27 years of age, when HRV parameters reach their maximum and become stable (Korkushko et al., 1991; Silvetti et al., 2001). A *p-*value of <0.05 was considered statistically significant.

## Results

### Somatic vs. Cognitive–Affective Symptoms and HRV

When considering the group as a whole, overall depressive symptoms were not associated with any of the autonomic measures (all *p*s > 0.31). In addition, HR, SDNN, rMSSD, NN50, HRV total power, and HF power were not predicted by cognitive–affective symptoms of depression (all *p*s > 0.67). Similarly, somatic symptoms of depression were unrelated to HR, SDNN, rMSSD, NN50, HRV total power, and HF power (all *p*s > 0.33).

### Somatic vs. Cognitive–Affective Symptoms and HRV in Individuals with vs. without Dysphoria

The groups did not differ in terms of sex (*p* = 0.46), with a predominance of female students both in the group with dysphoria (*n* = 23, 92%) and in the group without dysphoria (*n* = 31, 84%). The groups did not differ in terms of smoking habits: the current smokers were 6 (24%) and 18 (49%) in the group with and without dysphoria (*p* = 0.05), respectively. Similarly, the groups did not differ in terms of regular alcohol use (*p* = 0.39), with one (4%) and five (14%) regular alcohol drinkers in the group with and without dysphoria, respectively. The mean age was slightly higher in the group without dysphoria (*M* = 22.4, SD = 2.0) compared to the group with dysphoria (*M* = 21.2, SD = 1.8; *p* < 0.03). Overall depressive symptoms as well as cognitive–affective and somatic symptoms were significantly higher in the group with dysphoria (total BDI-II score, *M* = 15.8, SD = 4.0; cognitive–affective symptoms, *M* = 9.0, SD = 3.9; somatic symptoms, *M* = 6.8, SD = 1.8) than without dysphoria (total BDI-II score, *M* = 3.3, SD = 3.0; cognitive–affective symptoms, *M* = 1.2, SD = 1.4; somatic symptoms, *M* = 2.1, SD = 2.3; all *p*s < 0.001). Likewise, anxiety symptoms, as assessed with the STAI-Y2, were higher in the group with dysphoria (*M* = 53.6, SD = 9.4) than without dysphoria (*M* = 34.6, SD = 7.5, *p* < 0.001).

With respect to the group with dysphoria, the total BDI-II scores were not associated with any of the autonomic measures (all *p*s > 0.07). Linear regressions showed that somatic symptoms were significantly associated with SDNN, NN50, and HRV total power. Specifically, higher somatic symptoms were associated with lower SDNN, NN50, and HRV total power in the group with dysphoria. It is worth noting that these significant associations were independent of sex, anxiety symptoms, smoking habits, and regular alcohol use. By contrast, cognitive–affective symptoms were unrelated to SDNN, NN50, and HRV total power in the group with dysphoria. Both cognitive–affective and somatic symptoms were not associated with HR, rMSSD, and HF power. All the statistical details of the regression analysis in the group with dysphoria are reported in **Table 2**.

**Table 2:** Results of linear regression analysis with cognitive–affective or somatic symptoms predicting HRV parameters in the group with dysphoria.

HRV parameters	*B*	95% CI of B	SE *B*	β	*p*	*R*^2^
HR^*^						0.209
Cognitive–affective symptoms	0.986	-1.398 to 3.369	1.134	0.276	0.396	
Somatic symptoms	2.487	-1.531 to 6.505	1.913	0.327	0.210	
SDNN^*^						0.382
Cognitive–affective symptoms	-1.831	-5.509 to 1.847	1.751	-0.294	0.309	
Somatic symptoms	-6.321	-12.522 to -0.120	2.952	-0.476	0.046	
rMSSD^*^						0.203
Cognitive–affective symptoms	-1.896	-7.414 to 3.622	2.626	-0.230	0.480	
Somatic symptoms	-5.752	-15.055 to 3.552	4.428	-0.328	0.210	
NN50^*^						0.460
Cognitive–affective symptoms	-0.665	-9.191 to 7.861	4.058	-0.043	0.872	
Somatic symptoms	-16.398	-30.774 to -2.022	6.843	-0.498	0.028	
lnHRV total power^*^						0.417
Cognitive–affective symptoms	-0.088	-0.203 to 0.026	0.054	-0.442	0.123	
Somatic symptoms	-0.210	-0.403 to -0.017	0.092	-0.494	0.034	
lnHF^*^						0.318
Cognitive–affective symptoms	-0.020	-0.185 to 0.146	0.079	-0.074	0.805	
Somatic symptoms	-0.201	-0.480 to 0.078	0.133	-0.353	0.147	

^*^Model controlling for sex, anxiety symptoms, current smoking, and regular alcohol use. HRV, heart rate variability; HR, heart rate; SDNN, standard deviation of NN intervals; rMSSD, the square root of the mean of the sum of the squares of differences between adjacent NN intervals in ms; NN50, number of interval differences of successive NN intervals greater than 50 ms; lnHRV total power, log of heart rate variability total power in ms^2^; lnHF, log of high frequency power in ms^2^; B, non-standardized regression coefficient; SE B, standard error; β, standardized regression coefficient; 95% CI, 95% confidence interval.

With respect to the group without dysphoria, the total BDI-II scores were not associated with any of the autonomic measures (all *p*s > 0.12). Similarly, the cognitive–affective and somatic symptoms did not predict any of the HRV parameters. All the statistical details of the regression analysis in the group without dysphoria are reported in **Table 3**.

**Table 3:** Results of linear regression analysis with cognitive–affective or somatic symptoms predicting HRV parameters in the group without dysphoria.

HRV parameters	*B*	95% CI of B	SE *B*	β	*p*	*R*^2^
HR^*^						0.293
Cognitive–affective symptoms	0.925	-1.646 to 3.496	1.259	0.128	0.468	
Somatic symptoms	0.873	-0.644 to 2.390	0.743	0.199	0.249	
SDNN^*^						0.082
Cognitive-affective symptoms	0.900	-3.841 to 5.640	2.321	0.077	0.701	
Somatic symptoms	-0.351	-3.148 to 2.446	1.370	-0.049	0.799	
rMSSD^*^						0.050
Cognitive-affective symptoms	0.274	-4.704 to 5.253	2.438	0.023	0.911	
Somatic symptoms	0.330	-2.608 to 3.268	1.438	0.045	0.820	
NN50^*^						0.022
Cognitive-affective symptoms	1.601	-15.083 to 18.284	8.169	0.040	0.846	
Somatic symptoms	1.324	-8.521 to 11.168	4.820	0.055	0.785	
lnHRV total power^*^						0.111
Cognitive-affective symptoms	0.059	-0.149 to 0.267	0.102	0.113	0.567	
Somatic symptoms	-0.037	-0.159 to 0.086	0.060	-0.116	0.546	
lnHF^*^						0.084
Cognitive-affective symptoms	0.061	-0.226 to 0.349	0.141	0.087	0.666	
Somatic symptoms	0.032	-0.138 to 0.202	0.083	0.074	0.702	

^*^Model controlling for sex, anxiety symptoms, current smoking, and regular alcohol use. HRV, heart rate variability; HR, heart rate; SDNN, standard deviation of NN intervals; rMSSD, the square root of the mean of the sum of the squares of differences between adjacent NN intervals in ms; NN50, number of interval differences of successive NN intervals greater than 50 ms; lnHRV total power, log of HR variability total power in ms^2^; lnHF, log of high frequency power in ms^2^; B, non-standardized regression coefficient; SE B, standard error; β, standardized regression coefficient; 95% CI, 95% confidence interval.

## Discussion

The present study examined whether cognitive–affective and somatic symptoms of depression were differentially associated with time domain parameters of HRV in healthy individuals. Overall, self-reported depressive symptoms (i.e., the total BDI-II score) were not associated with any of the HRV parameters either when the group was considered as a whole or when participants were divided into a group with and a group without dysphoria. Similarly, no significant effects of cognitive–affective or somatic symptoms on any of the HRV parameters in the group as a whole or in individuals without dysphoria were noted. By contrast, SDNN, NN50, and HRV total power were significantly predicted by somatic, but not cognitive–affective, symptoms of depression in the group with dysphoria, while controlling for sex, anxiety symptoms, smoking habits, and regular alcohol use. Specifically, the somatic symptoms were inversely associated with SDNN, NN50, and HRV total power. Both somatic and cognitive–affective symptoms were unrelated to HR, rMSSD, and HF power in the group with dysphoria.

Our results are in line with previous findings showing an inverse association between somatic, but not cognitive–affective, symptoms of depression and HRV in patients with stable CAD (de Jonge et al., 2007). These novel findings add to the literature on the physiological mechanisms underlying the association between depression and cardiovascular diseases by showing that an inverse relationship between somatic depressive symptoms and HRV is present even in healthy individuals. Accordingly, the present data provide preliminary evidence that the relationship between somatic symptoms of depression and reduced HRV is independent of factors concomitant with cardiovascular diseases such as disease severity or the presence of comorbid medical conditions. Of note, the present study also showed that the relationship between somatic depressive symptoms and reduced HRV was independent of anxiety symptoms. The latter result is consistent with previous studies showing that depression, but not anxiety, is associated with reduced HRV (e.g., Pitzalis et al., 2001) and cardiovascular risk (e.g., Jiang et al., 2004). However, it should be noted that anxiety is comorbid with depression in more than 60% of cases (Kessler et al., 2005), and that anxiety rather than depression has been associated with reduced HRV in psychiatric or cardiovascular patients (e.g., Friedman, 2007; Martens et al., 2008). Clearly, future studies are warranted to examine the relative contribution of depression and anxiety to reduced HRV and cardiovascular risk in both clinical and non-clinical samples (Carney et al., 2000).

Our results are likely to explain why some studies did not find any association between overall depressive symptoms and reduced HRV in patients with CAD (Gehi et al., 2005) or post-MI (Martens et al., 2008). Likewise, the present results also provide an explanation for the inconsistent findings concerning the association between depressive symptoms and reduced HRV in psychiatric patients (e.g., Dallack and Roose, 1990; Yeragani et al., 1991; Rechlin et al., 1994; Moser et al., 1998). More specifically, the present findings are consistent with previous studies suggesting that depression is a heterogeneous disorder and that only specific subsets of symptoms may be related to poor cardiovascular health in individuals with dysphoria. Importantly, the finding that low HRV is associated with somatic symptoms of depression suggests that somatic rather than cognitive–affective symptoms are more likely to be implicated in the development of cardiovascular disease in medically healthy individuals with dysphoria. It is well-established, indeed, that reduced HRV reflects ANS dysfunctions, which, in turn, may predispose individuals with depression to ventricular tachycardia, ventricular fibrillation, myocardial ischemia, and sudden cardiac death (Carney et al., 2002, 2005). In line with this suggestion, somatic, but not cognitive–affective, symptoms of depression have been associated with low HRV (de Jonge et al., 2007), and with poor prognosis in cardiovascular patients (e.g., Watkins et al., 2003; de Jonge et al., 2006; Linke et al., 2009; Roest et al., 2011).

Along the same line of reasoning, it is important to note that somatic symptoms of depression were inversely related to SDNN of HRV. Among time domain parameters of HRV, the SDNN has been considered as one of the most accurate predictors of mortality in post-MI patients (Kleiger et al., 1987; Stein et al., 2000). Specifically, Kleiger et al. (1987) have reported that the risk of mortality is 5.3 higher in post-MI patients with SDNN < 50 ms compared to patients with SDNN > 100 ms. Clearly, longitudinal studies are warranted to examine whether SDNN may be a marker of the risk of cardiovascular diseases associated with somatic symptoms of depression.

Our results are also in line with previous findings by Bosch et al. (2009) showing that somatic symptoms of depression were related with low HRV in healthy preadolescents. However, some differences also exist between findings by Bosch et al. (2009) and those reported in the present study. First, while Bosch et al. (2009) found an inverse association between somatic symptoms of depression and HRV in the whole sample, we observed this relationship only in individuals with dysphoria (i.e., with a BDI-II score equal to or greater than 12 and at least two current depressive symptoms, at least 2 weeks in duration). An explanation that reconciles our findings with those by Bosch et al. (2009) is that the inverse relationship between somatic symptoms and HRV becomes much stronger when somatic symptoms exceed a threshold of severity. Second, in line with de Jonge et al.’s (2007), we observed that cognitive–affective symptoms were unrelated to HRV parameters, whereas Bosch et al. (2009) found an unexpected positive association between cognitive–affective symptoms and HRV parameters. This discrepant finding may be due to the different measurement instruments used. Specifically, while the BDI-II questionnaire reflects the criteria of the Diagnostic and Statistical Manual of Mental Disorders, Fourth Edition (DSM-IV; American Psychiatric Association, 1994), and accurately delineates cognitive and somatic depressive symptoms (Dozois et al., 1998), the Youth Self-Report (YSR; Achenbach and Rescorla, 2001) used by Bosch et al. (2009) was not developed to evaluate depressive symptoms according to the DSM-IV criteria. In addition, given that low internal consistency for both subscales of the YSR has been reported by Bosch et al. (2009), it is possible that the cognitive–affective scale of the YSR was heterogeneous, potentially biasing the relationship between cognitive–affective symptoms and HRV.

Although there is consistent evidence that increased sympathetic and/or parasympathetic cardiac control may represent one of the pathophysiological mechanisms linking depression to cardiovascular diseases, the relative contribution of the sympathetic and parasympathetic ANS to depression as a risk factor for cardiovascular diseases is still debated (Carney et al., 2001, 2005). Given that parasympathetic control exceeds sympathetic influence on the variability of resting HR time series (i.e., the HRV), the SDNN, as an estimate the overall HRV, is more likely to reflect cardiac vagal rather than sympathetic control (Task Force of the European Society of Cardiology, and the North American Society for pacing, and electrophysiology, 1996). In line with the de Jonge et al.’s findings (2007), our data suggest that somatic symptoms of depression may be associated with poor cardiac vagal control in medically healthy individuals with depressive symptoms. Consistent with our hypothesis, the NN50 count, which estimates HF variation in HR (Task Force of the European Society of Cardiology, and the North American Society for pacing, and electrophysiology, 1996), was also inversely associated with somatic symptoms of depression. However, given that rMSSD and HF power were unrelated to somatic symptoms in individuals with dysphoria, it remains unclear whether the inverse relationship between somatic symptoms and HRV is actually mediated by reduced cardiac vagal control (Bosch et al., 2009).

The present study has strengths and limitations. Individuals with cardiovascular illness were excluded from the present study, thus avoiding the potential confounding effects of cardiovascular disease on the association between somatic symptoms and reduced HRV. Also, this is the first study to our knowledge that examined the differential association between subsets of depressive symptoms and HRV while controlling for anxiety symptoms.

Some limitations to be noted include the relatively small sample size and, therefore, the fact that we did not conduct a factor analysis on our factor structure. However, the two-factor solution (cognitive–affective vs. somatic symptoms of depression) has been widely used in non-clinical (e.g., Whisman et al., 2000; Storch et al., 2004; Bosch et al., 2009) and clinical samples (e.g., Beck et al., 1996; Steer et al., 1998, 1999; de Jonge et al., 2007). Specifically, the two-factor solution in non-clinical samples (mostly students) usually consists of a first, cognitive–affective factor (Factor 1), and of a second, somatic factor (Factor 2; Beck et al., 1996; Whisman et al., 2000; Storch et al., 2004). In psychiatric patients, the somatic–affective factor is usually the first factor obtained and the cognitive factor is the second factor identified (Beck et al., 1996; Steer et al., 1998, 1999). Given that our study was focused on undergraduates with and without dysphoria, we adopted the two-factor solution with a cognitive–affective factor and a somatic factor (Carvalho Bos et al., 2009). In addition, in order to balance the number of items included in the two factors, we assigned each item either to cognitive–affective or somatic dimensions according to the two-factor structure obtained by Carvalho Bos et al. (2009). The high internal consistency of the cognitive–affective and somatic dimensions confirms the adequacy of the two-factor structure used in the present study. Second, most somatic symptoms of depression (e.g., fatigue, change in appetite, sleep problems) overlap with characteristics of vital exhaustion, a condition known to be associated with the risk for future coronary diseases (Appels et al., 1987). In addition, fatigue, which can be considered as one of the key symptoms of both depression and vital exhaustion or as a symptom by itself (Kop, 2012), has been consistently reported as a risk factor for cardiac diseases (e.g., McSweeney et al., 2003). Therefore, the association between somatic symptoms of depression and cardiac risk, as reflected by reduced HRV, may be confounded by vital exhaustion and/or fatigue. However, it should be noted that somatic depressive symptoms have been shown to predict mortality in patients with heart failure even after controlling for exertion fatigue (Smith et al., 2012). Targeted treatment studies and experimental designs are needed to examine the relative contribution of somatic depressive symptoms, vital exhaustion and fatigue to cardiac risk, as indicated by reduced HRV. Third, although we controlled for the effect of smoking status and alcohol use, other unhealthy life-style factors, such as poor dietary intake or physical inactivity, may confound the relationship between somatic depressive symptoms and reduced HRV (Kop, 2012). Future research should examine whether the association between somatic symptoms of depression and reduced HRV is independent of poor dietary intake and physical inactivity, which have been previously associated with reduced HRV (Puig et al., 1993; Mozaffarian et al., 2008). Finally, given that this study used cross-sectional data, causality could not be inferred. However, the present study aimed at examining whether time domain HRV parameters were differentially associated with somatic rather than cognitive–affective symptoms of depression, but we do not claim that reduced HRV is either the cause or the result of cognitive–affective and/or somatic depressive symptoms. Clearly, further research should specifically address this issue.

Based on the present findings, future studies aimed at examining the relationship between depression and reduced HRV in cardiovascular patients should take into account the differential impact of cognitive–affective and somatic symptoms on ANS functioning. From a clinical point of view, the present study suggests that somatic rather than cognitive–affective symptoms of depression are more likely to be implicated in the depression-related risk of developing cardiovascular diseases in medically healthy individuals with dysphoria. The longitudinal evaluation of subsets of depressive symptoms, HRV parameters, and cardiovascular health is crucial to determine whether a reduced SDNN associated with somatic symptoms may predict the onset of cardiovascular disease in medically healthy individuals with depressed mood.

## Conflict of Interest Statement

The authors declare that the research was conducted in the absence of any commercial or financial relationships that could be construed as a potential conflict of interest.
